# Clathrin inhibitor Pitstop-2 disrupts the nuclear pore complex permeability barrier

**DOI:** 10.1038/srep09994

**Published:** 2015-05-06

**Authors:** Ivan Liashkovich, Dzmitry Pasrednik, Valeria Prystopiuk, Gonzalo Rosso, Hans Oberleithner, Victor Shahin

**Affiliations:** 1 Institute of Physiology II, Robert-Koch-Str. 27b, 48149 Münster, Germany

## Abstract

Existence of a selective nucleocytoplasmic permeability barrier is attributed to Phenylalanine-Glycine rich proteins (FG-nups) within the central channel of the nuclear pore complex (NPC). Limited understanding of the FG-nup structural arrangement hinders development of strategies directed at disrupting the NPC permeability barrier. In this report we explore an alternative approach to enhancing the NPC permeability for exogenous macromolecules. We demonstrate that the recently discovered inhibitor of clathrin coat assembly Pitstop-2 compromises the NPC permeability barrier in a rapid and effective manner. Treatment with Pitstop-2 causes a collapse of the NPC permeability barrier and a reduction of Importin β binding accompanied by alteration of the NPC ultrastructure. Interestingly, the effects are induced by the same chemical agent that is capable of inhibiting clathrin-mediated endocytosis. To our knowledge, this is the first functional indication of the previously postulated evolutionary relation between clathrin and NPC scaffold proteins.

Permeability barrier of the NPC^1^ has been considered to be the result of a certain mode of interaction among the Phenylalanine-Glycine (FG)-repeat proteins^2^. Therefore, the effort directed towards reducing the stringency of the barrier has been primarily targeted at disrupting the interactions between the FG-repeats. The goal was achieved by applying high concentrations of aliphatic alcohols^3^,^4^. This approach has later been demonstrated to cause dissociation of NPC components^5^. Moreover, toxicity and moderate effect on intranuclear transgene delivery in cell culture^6^ and no effect *in vivo*^7^ have questioned the utility of this strategy of NPC permeabilization. Therefore we considered an alternative strategy. We reasoned that disassembly of the NPC during mitosis^8^ clearly indicates that increasing the NPC permeability by disrupting the NPC scaffold should also be feasible. The choice of the active agent was dictated by the previously proposed common ancestral coatomer origin of clathrin and several of the NPC scaffold proteins^9^. Based on this assumption we searched for the candidates among small molecule inhibitors of the clathrin coat assembly. The most promising among them was the recently developed Pitstop-2^10^. Specific binding of Pitstop-2 to the β-propeller N-terminal domain (NTD) of clathrin heavy chain has been demonstrated by resolving the atomic structure of the complex^10^. As a result of this direct binding the drug was able to inhibit the clathrin coat assembly and the uptake of classical substrates of clathrin-mediated endocytosis^10^. Direct interaction of Pitstop-2 with the β-propeller domain of clathrin prompted us to test the following hypothesis. If the structural resemblance between clathrin and the β-propeller nucleoporins is a consequence of a conserved function, then Pitstop-2 might be able to disrupt the NPC scaffold thereby leading to a breakdown of the NPC permeability barrier.

## Results

**Figure 1:**
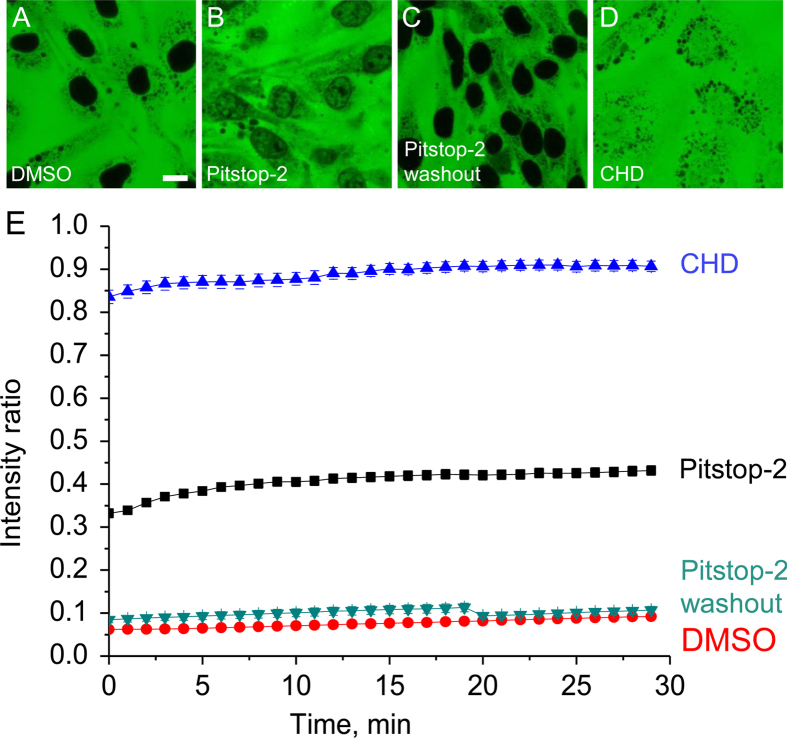
Pitstop-2 induces reversible collapse of the NPC permeability barrier Representative confocal images of digitonin-permeabilized GM7373 cells at 30 minutes after treatment with 0.1% DMSO (**A**), 30 μM Pitstop-2 (**B**) and 5% CHD (**D**). Washout of Pitstop-2 one hour prior to permeabilization leads to restoration of permeability barrier (**C**). Scale bar = 10 μm. Kinetics of 70 kDa dextran influx into the nuclei of digitonin-permeabilized cells upon treatment with respective compounds (**E**). 20 cells from three separate experiments were analyzed for each condition. Error bars represent standard error of the mean.

To demonstrate the ability of Pitstop-2 to disrupt the NPC barrier function we used the digitonin-permeabilized cell assay^11^. Ea.hy 926 human umbilical vein endothelial cell line^12^ and GM 7373 bovine aortic endothelial cell line^13^ were tested. Treatment of the cultured cells with 30 μM Pitstop-2 upon permeabilization with digitonin has resulted in a robust influx of the 70 kDa FITC dextran into the nuclei (Fig. 1B,E), although somewhat less efficiently than the influx induced by trans-1,2-cyclohexanediol (CHD) (Fig. 1D,E), which we used as a benchmark of the NPC permeabilization. However, it is important to note that the effective concentration of Pitstop-2 is more than four orders of magnitude lower than that of CHD (30 μM vs. 430 mM or 5%). Several control experiments were performed to ensure that the effect of Pitstop-2 is specific and is not caused by a defect of the nuclear envelope lipid bilayer. Combining FITC-labeled 250 kDa dextran^14^ with TRITC-labeled 65–85 kDa dextran has revealed that only the 65–85 kDa TRITC dextran exhibits accelerated diffusion into the nucleus in presence of Pitstop-2 while 250 kDa FITC dextran remains excluded from the nucleoplasm unless the lipid component of the nuclear envelope is compromised by 1% Triton X100 (Fig. S1). Treatment with a Pitstop-2 negative control compound which is structurally highly similar to Pitstop-2 (Abcam, ab120688) was unable to induce the influx of 65–85 kDa dextran similarly to the effect of 0.1% DMSO alone used as a solvent (Fig. S1). To test if the NPC permeabilization effect by Pitstop-2 can be attributed to an indirect action via clathrin we examined whether another clathrin inhibitor Pitstop-1, which binds to the β-propeller domain, is able to induce a similar effect. However, Pitstop-1 was unable to induce the influx of 70 kDa FITC dextran into the nuclei (Fig. S2), strongly indicating that permeabilization of the NPC induced by Pitstop-2 is independent of clathrin. The effect of Pitstop-2 was reproducible irrespective of the time point of its application. Incubation with Pitstop-2 30 minutes before digitonin permeabilization resulted in a similar effect as the treatment of the cells that have already been permeabilized (Fig. S3). To demonstrate the reversibility of the effect, Pitstop-2 was washed out one hour prior to permeabilizing the cells with digitonin. Washout of Pitstop-2 has demonstrated an interesting discrepancy between the two cell lines. While GM7373 bovine aortic endothelial cells were able to restore the barrier within 60 minutes of the Pitstop-2 washout (Fig. 1C,E), Ea.hy 926 human endothelial cells were unable to restore their barrier function even after 24 hours after Pitstop-2 washout (Fig. S4). Taken together, the results of the permeabilization experiments show that Pitstop-2 is capable of facilitating diffusion of large macromolecules that are normally excluded from the nucleus.

**Figure 2:**
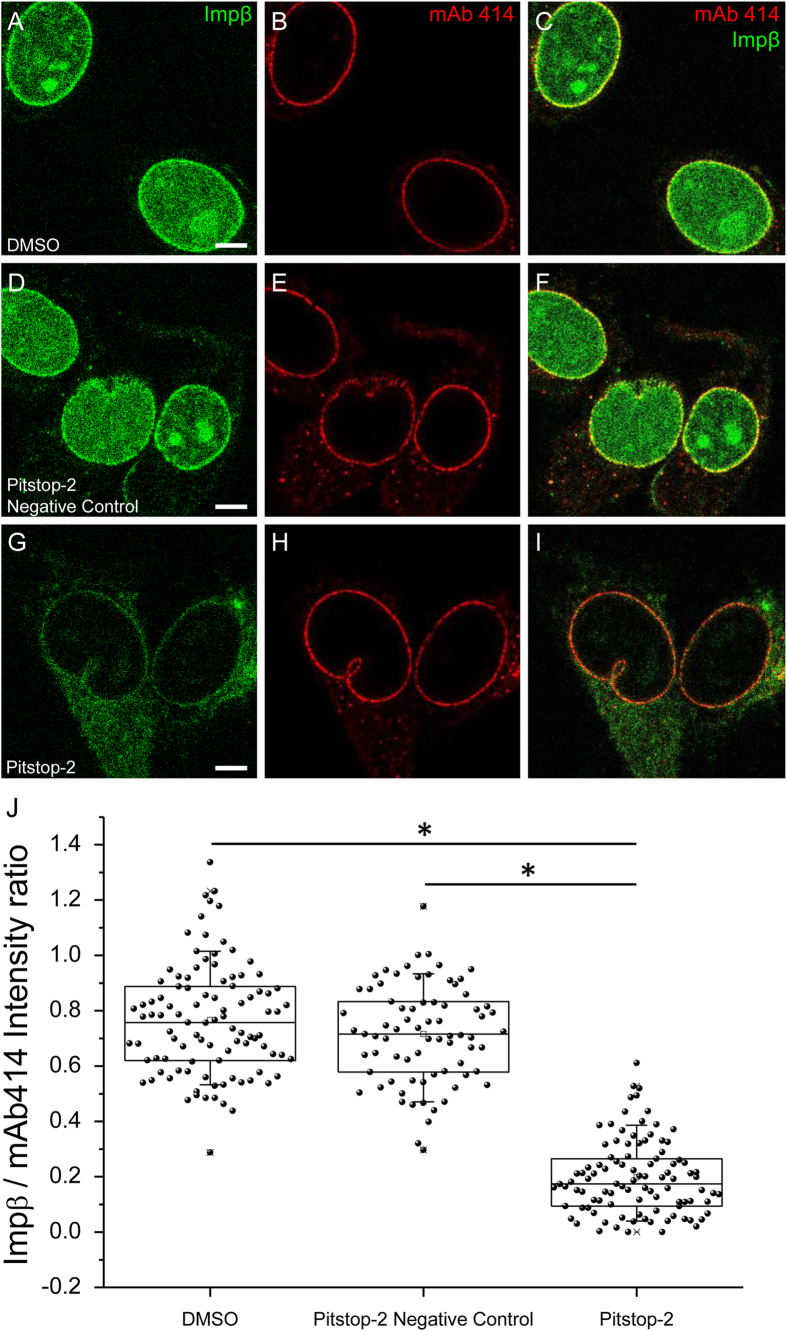
Pitstop-2 impairs binding of Impβ to the NPCs Binding of Alexa-488 Impβ to the NPCs in presence of 0.1% DMSO (**A**), 30 μM Pitstop-2 negative control (**D**) and 30 μM Pitstop-2 **(G)** was examined with confocal microscopy. Concomitant immunostaining with mAb414 (**D**, **E**, **H**) was used to confirm the specificity of the Impβ binding to the NPCs (**C**, **F**, **I**). Scale bar = 5 μm. mAb414 staining was not affected by any of the investigated compounds and was therefore used as normalization control to quantify the extent of Impβ binding inhibition by Pitstop-2 (**J**). 100 individual NPCs from 20 different cells were analyzed for each condition. Statistical significance of the differences was assessed by Mann-Whitney test (nonparametric groups) at p ≤ 0.05. Quantification approach is further detailed in Fig. S5

To investigate whether the Pitstop-2-induced permeabilization of the NPC has an impact on the NPC interaction with the major active transport receptor Importin β (Impβ), we incubated the digitonin-permeabilized mammalian cells with the bacterially expressed and Alexa-488-labeled Impβ^15^. To illustrate the specificity of the Impβ binding we performed a concomitant immunostaining with mAb414, a monoclonal antibody which specifically binds several FG nucleoporins^16^. As a reslut, we were able to quantify the extent of Impβ binding to the NPCs in presence of Pitstop-2 using the antibody signal as a normalization control. Confocal laser scanning microscopy has revealed that Impβ was not only able to bind to the NPCs of the digitonin-permeabilized Ea.hy926 but also accumulated in the nucleoplasm in presence of DMSO or the Pitstop-2 negative control compound (Fig. 2A–F). In presence of Pitstop-2 the situation was markedly different (Fig. 2G–I). The binding of Impβ to the NPCs was dramatically reduced while the mAb414 signal remained largely unaffected. Moreover, the intranuclear accumulation of Impβ was largely abolished. To quantify the reduction of the Impβ binding we analyzed the intensity profiles of 100 NPCs of 20 different cells from two separate experiments. Peak intensity values of Impβ were normalized against the peak intensity values of mAb414 intensity profiles as specified in Fig. S5. Plotting the Impβ/mAb414 intensity ratio values has revealed a significant drop of the Impβ binding to the NPCs only in presence of Pitstop-2. The Pitstop-2 negative control compound had no significant effect on Impβ binding to the NPCs of Ea.Hy926 endothelial cells similarly to its lack of effect on the NPC permeability. A similar reduction in Impβ binding was observed in GM7373 bovine endothelial cells (Fig. S6). In Ea.hy 926 endothelial cells the effect was dependent on Pitstop-2 concentration and peaked between 3 μM and 30 μM (Fig. S7). The observed reduction of the Impβ binding is a strong indication that Pitstop-2 interferes directly with the NPCs. It also signifies that the reduced binding of Impβ could lead to an impairment of the active nucleocytoplasmic transport in living cells.

**Figure 3:**
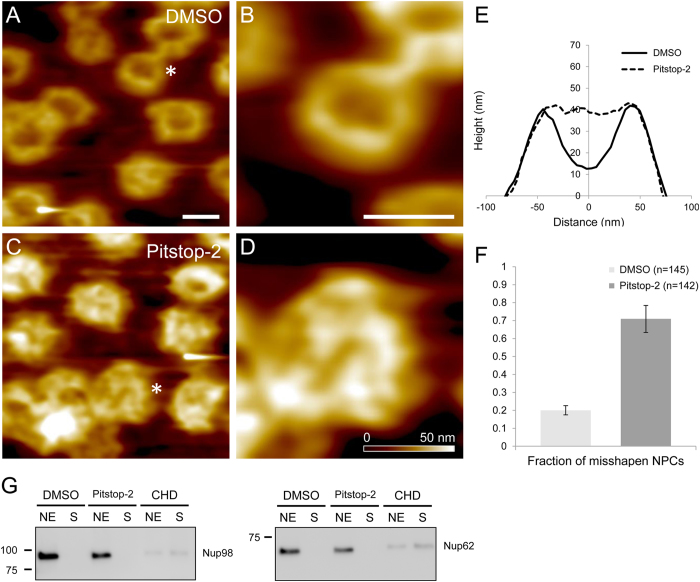
Pitstop-2 permeabilizes NPCs by disrupting the NPC ultrastructure without dissociating putative barrier-forming FG-nups AFM images of *Xenopus laevis* nuclear envelopes treated with DMSO (**A**, **B**). Typical appearance of the intact NPCs (**A**), asterisk marks an NPC magnified in panel (**B**). AFM images of *Xenopus laevis* nuclear envelopes treated with Pitstop-2 (**C**, **D**). Magnified image of an NPC marked by asterisk in panel (**D**). Scale bars = 100 nm. Mean section profile of 80 DMSO-treated NPCs overlaid with the mean section profile of 80 Pitstop-2-treated NPCs demonstrate an occlusion of the NPC central channel in response to Pitstop-2 treatment (**E**). Morphometric quantification of DMSO-treated NPCs vs. Pitstop-2-treated NPCs demonstrate a drastic increase in the fraction of misshapen NPCs in Pitstop-2-treated preparations (**F**). Channel depth of >10 nm was used as a criterion to differentiate between an intact or misshapen NPC. Western-blots of *Xenopus leavis* nuclear envelopes against putative barrier-forming FG-nups (**G**) show no detectable Pitstop-2-induced dissociation of either Nup62 or Nup98 (NE – nuclear envelope; S – supernatant). Imaging experiments were performed using oocytes from two different animals in duplicates. Western blot samples were prepared from two different animals in triplicates.

To determine whether Pitstop-2 directly interferes with the NPC ultra-structure, we performed atomic force microscopy (AFM) imaging of the nuclear envelopes mechanically isolated from *Xenopus laevis* oocytes^17^ and subjected to Pitstop-2 treatment. AFM imaging has demonstrated that treatment with Pitstop-2 strongly interferes with the ultrastructural organization of the NPCs (Fig. 3). To quantify the structural changes we performed cross-sectional analysis of Pitstop-2-treated NPCs and compared it to DMSO control (Fig. 3E) as described previously^5^. Averaging cross-sections of 80 NPCs for each of the experimental conditions has shown that while the control NPCs retain the overall shape with a pronounced central channel, the Pitstop-2 treated NPCs exhibit an occlusion of the central channel. We used the channel depth of >10 nm as a criterion to discriminate between the intact NPCs and the NPCs with a compromised structure. Such analysis has demonstrated that while the DMSO-treated nuclear envelopes have about 20% of misshapen NPCs, their fraction increases to ~80% after treatment with Pitstop-2 (Fig. 3F). The occlusion of the NPC central channel is particularly surprising if we consider the fact that Pitstop-2 induces a breakdown of the NPC permeability barrier in mammalian cells. The structural alterations induced by Pitstop-2 are also markedly different from those induced by CHD^5^ which primarily affects the nucleoporins residing in the NPC central channel by dissociating them from the NPC. We therefore examined whether Pitstop-2 is able to dissociate the putative barrier-forming nucleoporins Nup62^5^ or Nup98^18^. Consistently with the lack of Pitstop-2 effect on mAb414 immunostaining described in the previous section neither of these nucleoporins was detected in the supernatants of the Pitstop-2 treated nuclear envelopes. (Fig. 3G; Fig. S7). Biochemical extraction of 100 manually isolated nuclear envelopes with Pitstop-2 and partial proteomic analysis of the supernatant (Fig. S9) did not reveal any dissociation of the NPC components upon Pitstop-2 application. Ultrastructural AFM data together with the partial biochemical analysis of Pitstop-2- treated nuclear envelopes indicate that the effect of Pitsop-2 on NPCs is markedly different from that of CHD and largely independent of dissociation of FG-nucleoporins.

## Discussion

In this report we show that the recently developed small molecule clathrin inhibitor Pitstop-2 induces robust structural and functional alterations of NPCs of three different species of vertebrates (*Bos taurus*, *Homo sapiens* and *Xenopus laevis*). Comprehensive analysis of the NPC functionality subjected to Pitstop-2 treatment was performed. It included assessment of the permeability barrier integrity, binding of Impβ as an indicator of the active transport as well as AFM imaging of the Pitstop-2-induced changes of the NPC ultrastructure.

To evaluate the ability of Pitstop-2 to compromise the barrier function of the NPCs, we compared its barrier disrupting activity with that of the previously described disruptor of the NPC permeability barrier CHD^3^. We found that Pitstop-2 is able to reliably induce a comparable breakdown of the nucleocytoplasmic barrier in the digitonin-permeabilized cell system at much lower concentrations (30 uM vs. 430 mM) than CHD. The results of the control experiments with 250 kDa dextran and Pitstop-2 negative control compound largely eliminate the possibility of Pitstop-2-induced disruption of the nuclear envelope lipid component. No enhancement of permeability in presence of Pitstop-1 speaks against the possibility of an indirect clathrin-mediated effect.

The effect can be exerted before permeabilization with digitonin as is evident from the washout experiments. This is a strong indication that the breakdown of the NPC permeability barrier by Pitstop-2 can be induced in living cells as well. An important characteristic of the NPC permeability barrier disruptor is its ability to act reversibly. Interestingly, the extent of reversibility was markedly different in bovine vs. human cell lines. While GM7373 bovine endothelial cells were able to restore the barrier within one hour after Pitstop-2 washout, Ea.hy 926 human endothelial cells were unable to recover even after 24 hours of incubation in Pitstop-2 free medium. We initially hypothesized that the previously demonstrated^19^ increased toxicity of Pitstop-2 for cancerous cells can be linked to their inability to restore the nucleocytoplasmic barrier. Since the Ea.hy 926 human endothelial cell line originates from a fusion of human umbilical vein endothelial cells and lung carcinoma cells^12^ it could have retained the cancerous phenotype that makes it susceptible to Pitstop-2. However, permeability experiments with non-cancerous primary umbilical vein endothelial cells have also resulted in a lack of nucleocytoplasmic barrier recovery upon the washout of Pitstop-2 (data not shown). Therefore, our current interpretation of this discrepancy between the human and bovine cell lines is that Pitstop-2 has a higher affinity to its target within the NPC in human cells than in bovine cells. As a result, it prevents the restoration of the nucleocytoplasmic permeability barrier even 24 hours after the removal of the drug from the surrounding medium. An alternative explanation would be the tissue-specific variations in nucleoporin expression that could potentially be targeted by Pitstop-2^20^.

The second functional readout of the NPCs is its ability to carry out active transport. One of the first steps of this process is believed to be mediated by the interaction of the major transport receptor Impβ with the FG-nucleoporins within the NPC central channel^21^. Therefore, we monitored the binding of Impβ sparsely labeled with Alexa-488 (~2.4 molecules per one molecule of Impβ) to the NPCs of permeabilized mammalian cells in presence and in absence of Pitstop-2. Additional staining of the NPCs with the mAb414 has enabled quantitative assessment of the Pitstop-2 effect on the Impβ binding to the NPCs of the human endothelial cells. In both bovine and human cell lines Pitstop-2 was able to decrease the ability of Impβ to bind to the NPCs implicating an impaired active transport as well. Since we were unable to detect dissociation of the two major FG-nucleoporins implicated in permeability barrier establishment and maintenance from the *X. laevis* nuclear envelopes further supported by the lack of change in mAb414 staining of the mammalian NPCs, we interpret the reduced levels of Impβ binding as a consequence of the structural alterations of the NPCs induced by the Pitstop-2. It appears that the simple presence of FG-nucleoporins is not sufficient for efficient binding of the Impβ to the NPCs and its accumulation within the intranuclear space. Precise spatial arrangement of FG-nucleoporins that interact with the Impβ seems to be just as important. If the effect of Pitstop-2 on the active transport can be reproduced *in vivo,* the drug will turn out to be an interesting example of an antiviral agent which can not only inhibit the viral uptake but also hinder the passage of subviral structures into the nucleus.

**Figure 4:**
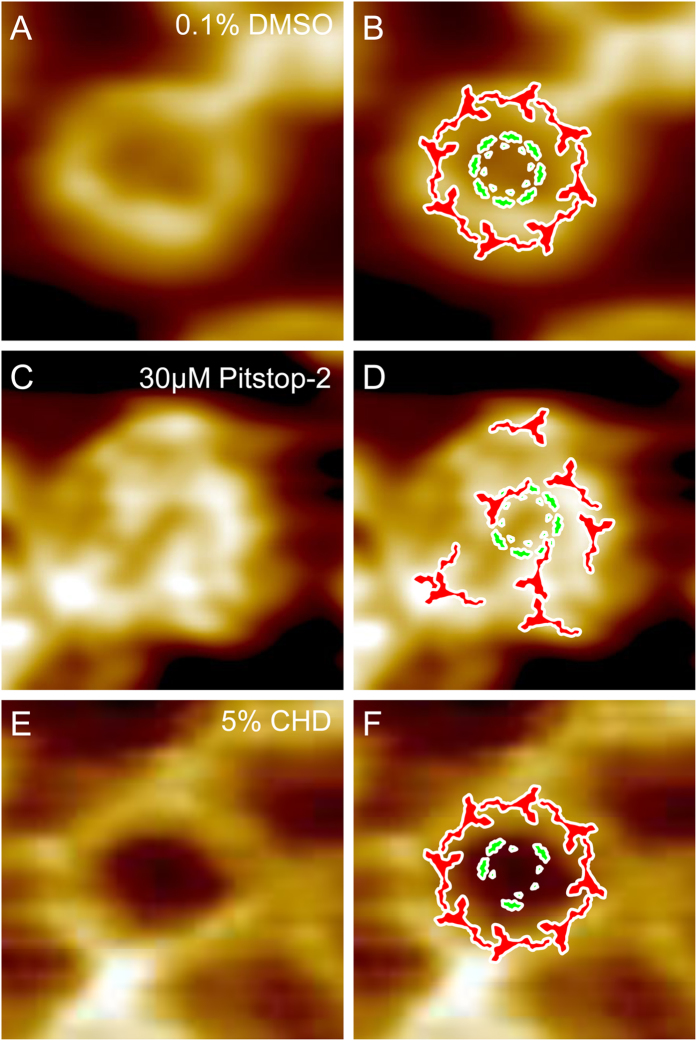
Hypothetical model of the mechanism of the NPC permeability barrier disruption by Pitstop-2 AFM image of an intact NPC (**A**) overlaid with schematic representations of Nup107–160 subcomplexes (red) and nucleoporins residing within the NPC central channel (green) (**B**). NPCs treated with 30 μM Pitstop-2 display an occluded central channel and an array of substructures very closely resembling the Y-shaped Nup107–160 subcomplexes in both shape and size (**C**, **D**). The structural arrangement of the FG-nucleoporins cannot be derived from our AFM data. However, we do not detect FG-nucleoporin dissociation from the Pitstop-2-treated NPC from either the *X. laevis* or mammalian nuclei. On the other hand, the barrier-disrupting effect of CHD (**E**, **F**) is targeted at the FG-nucleoporins residing within the NPC central channel and is based on dissociation of FG-nucleoporins (**F**).

Increased nuclear envelope permeability for 70 kDa dextran together with the reduction of Impβ binding provide strong evidence in support of the initial hypothesis that Pitstop-2 can serve as a disruptor of the NPC permeability barrier. However, the proposed mechanism of the NPC permeabilization through the disruption of the NPC scaffold needed to be tested on the level of a single NPC ultrastructure. For this AFM imaging of Pitstop-2-treated nuclear envelopes isolated from the oocytes of *X. laevis* was performed. The evolutionary distance between the mammalian cells and the frog oocyte might be a limitation. However, the advantages of using *X. laevis* oocyte nuclear envelope significantly outweigh the limitations. Manual isolation of the nuclei and nuclear envelope preparation for imaging ensures that the drug effect is direct and is unlikely to be mediated by any cytoplasmic or nucleoplasmic components. AFM imaging revealed that the mechanism of the NPC permeabilization by Pitstop-2 is very different from that of CHD. While CHD removes progressively increasing quantities of nucleoporins which occupy the NPC central channel^5^, Pitstop-2 seems to occlude the NPC central channel without inducing any detectable dissociation of the putative barrier-forming nucleoporins Nup62 or Nup98. The unexpected lack of nucleoporin dissociation indicates that Pitstop-2 disrupts a very limited number of nucleoporin-nucleoporin interactions, if not a single one. However, this interaction is critical for maintaining both the passive permeability barrier function of the NPC and its ability to interact with Impβ. We believe that Pitstop-2 disrupts the head-to-tail arrangement of the Y-complexes by interfering with the interactions between the β-propeller proteins. As a result, the Y-complex is probably pulled into the lumen of the NPC central channel by the still intact interactions among the FG repeats causing the occlusion of the central channel (Fig. 4). We think that Pitstop-2 targets an intersubcomplex interaction that involves the β-propeller nucleoporins. Although the β-propeller targeting within the NPC by Pitstop-2 remains to be confirmed, it is in agreement with the role of the β-propeller proteins as the assembly hubs for the NPC scaffold as postulated by Bui *et al*^22^.

Taken together, we were able to demonstrate a novel strategy of the NPC permeabilization by a small molecule inhibitor of clathrin Pitstop-2. The mechanism of the NPC permeability barrier breakdown induced by Pitstop-2 seems to be fundamentally different from the most of the previously reported mechanisms in that it does not require dissociation of the NPC components. It is unlikely that application of Pitstop-2 in its current form will provide a dramatic increase of intranuclear delivery of therapeutic DNA due to its inhibition of endocytosis. However, it can be a promising lead substance for designing the second generation of the NPC permeability barrier-disrupting chemical agents because of its low active concentration compared to CHD, broad species targeting, mild and in some cases reversible permeabilization. To our knowledge, this report is the first to provide a functional indication of the previously postulated structural relatedness of the clathrin coat and the NPC scaffold proteins. Further exploration of this notion might reveal additional unifying themes and foster better understanding of these two areas of cell biology.

## Methods

### Nuclear barrier permeability and Impβ binding to the NPCs of Pitstop-2-treated mammalian cells

Experiments were performed as described previously^23^ with the following modifications. GM 7373 and Ea.hy 926 endothelial cells were cultured at 37 °C, 5% CO2 and 100% humidity in minimal essential medium containing 1% non-essential amino acids, 1% MEM vitamins (Invitrogen Corp., Karlsruhe, Germany) and 10% fetal calf serum (FCS, PAA Clone, Coelbe, Germany). For imaging, cells were cultured on glass bottom petri dishes (WillCo Wells B. V., Amsterdam, Netherlands). Pitstop-2 (Abcam, Cambridge, UK) and Pitstop-2 negative control compound were used as provided by the manufacturer without further purification. 30 mM Pitstop-2 stock in DMSO was diluted to specified concentrations in FCS-free medium and was added 30 minutes prior to digitonin permeabilization or dissolved in Transport Buffer (TB, 20 mM HEPES, 110 mM K-Acetate, 5 mM Na-Acetate, 2 mM Mg-Acetate, 1 mM EGTA (pH 7.3), 2 mM DTT) and added directly after permeabilization. For reversibility experiments, washout of Pitstop-2 was achieved by replacing the Pitstop-2 dissolved in FCS-free medium with FCS-containing medium without the Pitstop-2 for a specified period of time. Permeabilization was performed by 20 μg/ml digitonin solution in TB for 5 minutes. Upon permeabilization, buffer was replaced with digitonin-free TB containing 200 μg/ml 70 kDa FITC dextran (Sigma-Aldrich, Steinheim, Germany) for investigating the integrity of the nucleocytoplasmic barrier. Images were taken in the mid-plane of the nuclei using Leica SP8 confocal laser scanning microscope equipped with hybrid detection system for photon counting (Leica, Wetzlar, Germany) at a rate of one image per minute for 30 minutes. Dynamics of the FITC-dextran influx was then evaluated by determining the ratio between the intranuclear fluorescence intensity and the extracellular background intensity.

For investigating the impact of Pitstop-2 on the Impβ binding to the NPCs, cells were permeabilized with digitonin as described above. After permeabilization with digitonin immunostaining with mAb414 was performed using 1:100 dilution of the primary antibody and 1:200 dilution of the secondary Alexa-568-labeled anti-mouse antibody (Life Technologies GmbH, Darmstadt, Germany). Subsequently, immunostained cells were treated with 30 μM Pitstop-2 in TB. For confocal imaging, Pitstop-2-containing TB was replaced with TB supplemented with 0.13 μM of Alexa-488-labeled Impβ. Impβ was expressed as described previously^15^ and labeled with Alexa Fluor 488 protein labeling kit (Life Technologies, Darmstadt, Germany) according to the instructions of the manufacturer so that each Impβ molecule was labeled with about 2.4 Alexa molecules as determined spectrophotometrically. Confocal imaging was performed 10 minutes after addition of the labeled Impβ. Fluorescence intensity of individual NPCs was analyzed by section profiling and determining the peak intensity.

### *Xenopus laevis* nuclear envelope preparation for AFM imaging

All experimental procedures on *X. laevis* and oocytes were approved by the local veterinary office (Project number 8.87-51.05.20.10.027). Nuclear envelopes were prepared essentially as described previously^5^ with the following modifications. Nuclei isolated from stage VI *X. laevis* oocytes were incubated in nuclear isolation medium (NIM, 87 mM NaCl, 3 mM KCl, 1.5 mM CaCl_2_, 1 mM MgCl_2_, 10 mM HEPES, pH 7.4) supplemented with 30 μM Pitstop-2 for 10 minutes. Nuclear envelopes were prepared and fixed with 1% glutaraldehyde solution in NIM supplemented with 30 μM Pitstop-2.

Multimode atomic force microscope (AFM) (Bruker AXS, Mannheim, Germany) was used for investigating the impact of Pitstop-2 on the NPC ultrastructure. Contact mode topographic imaging using OMCL-TR400PSA-3 probes (Atomic Force F&E GmbH, Mannheim, Germany) at 256 scan lines per area with a scan rate of 1.5 Hz was performed. Images were processed using Scanning Probe Image Processing software (SPIP, Image Metrology, Lyngby, Denmark).

### Pitstop-2 extraction of *X. laevis* nuclear envelopes

Extraction of nuclear envelopes with Pitstop-2 or CHD for detection of Nup dissociation was performed as described previously^5^. Ten nuclear envelopes per western blot sample were isolated and incubated for 30 minutes in NIM supplemented with 30 μM Pitstop-2, 5% CHD (Sigma-Aldrich) or 0.1% DMSO. Supernatant was aspirated from the nuclear envelopes which were subsequently solubilized with 5% SDS. Proteins contained in both fractions were precipitated by acetone and solubilized in Laemmli buffer. mAb414 (Covance, Münster, Germany) and anti-Nup98 (generous gift of Dr. Wolfram Antonin) at 1:1000 dilution were used for detecting Nup62 and Nup98, respectively.

For proteomic analysis of Pitstop-2 extract, 100 nuclear envelopes were isolated and extracted with Pitstop-2 as described above. After protein precipitation of both the nuclear envelope and supernatant fractions, solubilized proteins were separated using standard SDS-PAGE and stained with Coomassie brilliant blue (Sigma-Aldrich). Protein bands detected in the supernatant fraction were identified at the Center of Integrated Functional Genomics (IFG, Münster, Germany).

### Statistical data analysis

Statistical analysis was performed using Sigma Plot software (Systat Software GmbH, Erkrath, Germany). Statistical significance of the observed differences was tested using a t-test for the normally distributed data and Mann-Whitney signed rank test for the data that was not normally distributed. Compared groups were considered as significantly different if p ≤ 0.05.

## Author Contributions

I.L., D.P., V.P. and V.S. designed the experiments; I.L., D.P., V.P., G.R. and V.S performed the experiments; V.P. and D.P. performed statistical analysis of the fluorescence imaging data; I.L., V.P., H.O. and V.S. wrote and edited the manuscript.

## Additional Information

**How to cite this article**: Liashkovich, I. *et al.* Clathrin inhibitor Pitstop-2 disrupts the nuclear pore complex permeability barrier. *Sci. Rep.* **5**, 09994; doi: 10.1038/srep09994 (2015).

## Supplementary Material

Supplementary Figures
