# Supplementary material for: Clathrin inhibitor Pitstop-2 disrupts the nuclear pore complex permeability barrier
Source: Sci Rep. 2015 May 6;5:9994. doi: 10.1038/srep09994 (PMC4421796; doi:10.1038/srep09994)
Supplement: Supplementary Figures [file srep09994-s1.pdf]

Supplementary information

Clathrin inhibitor Pitstop-2 disrupts the nuclear pore complex permeability barrier

*Ivan Liashkovich[1†], Dzmitry Pasrednik[1†], Valeria Prystopiuk[1], Gonzalo Rosso[1], Hans Oberleithner[1] and Victor Shahin[1]**

[1]Institute of Physiology II, Robert-Koch-Str. 27b, 48149 Münster, Germany

Revised Version

SREP-14-11344-T

*Corresponding Author:

Victor Shahin

Institute of Physiology II, University of Münster, Robert-Koch Str. 27b, 48149 Münster, Germany

Tel.: +492518355327

E-mail: shahin@uni-muenster.de

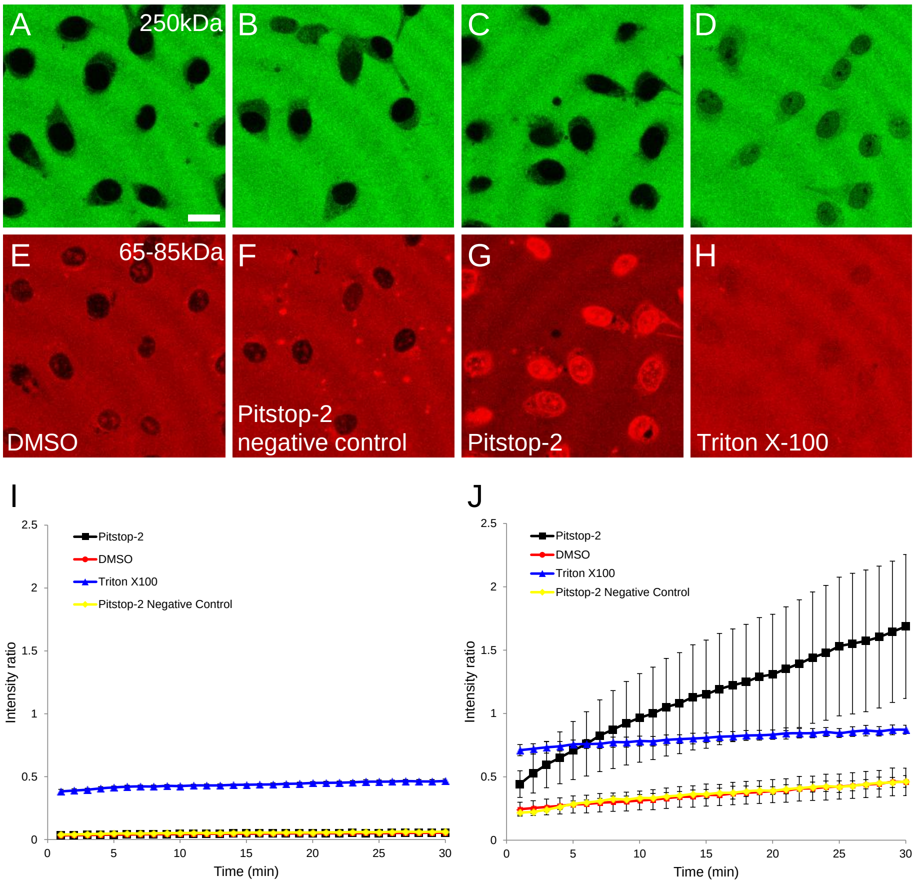

Figure S1|Pitstop-2 accelerates diffusion of 65-85kDa dextran but not of 250kDa dextran. Representative confocal images of digitonin-permeabilized Ea.Hy926 cells at 30 minutes after treatment with 0.1% DMSO **(A, E)**, 30µM Pitstop-2 negative control compound **(B, F)**, Pitstop-2 **(C, G)** and Triton X100 **(D, H)**. Quantification of the 250kDa dextran influx into the nuclei at conditions mentioned above demonstrate a significant influx of the large dextran only in response to 1% Triton X100 treatment **(I).** Smaller dextran (65-85kDa), however, exhibits an influx and even accumulation within the nuclei in presence of Pitstop-2, but not in presence of DMSO or the Pitstop-2 negative control compound **(J)**. Scale bar = 20µm. 21 cells from three separate experiments were analyzed for each condition. Error bars represent standard error of the mean.

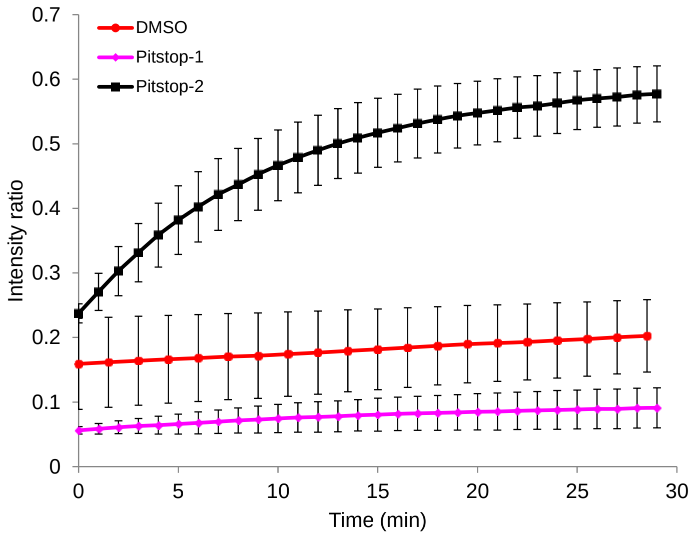

Figure S2|Pitstop-1 is unable to permeabilize the NPCs for 70kDa dextran. Significant increase of the dextran influx into the nuclei is detected only in presence of Pitstop-2 but not in presence of Pitstop-1 or DMSO. 20 cells from three separate experiments were analyzed for each condition. Error bars represent standard error of the mean.

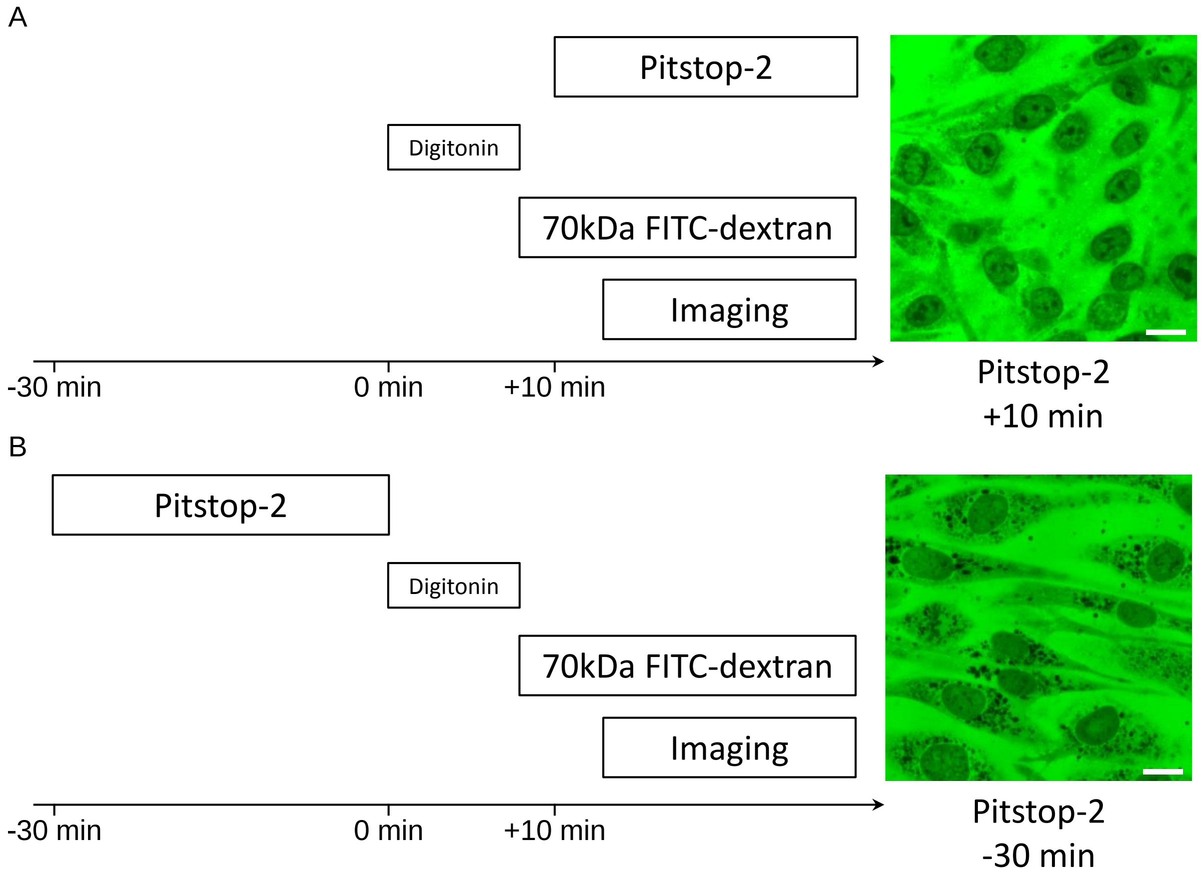

Figure S3|Pitstop-2 induces collapse of the NPC permeability barrier irrespective of the time of treatment. Pitstop-2 induces NPC permeability barrier collapse when added directly to digitonin-permeabilized cells **(A)**. Pitstop-2 leads to the collapse of the NPC permeability barrier when added 30 minutes prior to digitonin permeabilization but excluded from the experiment after permeabilization **(B)**.

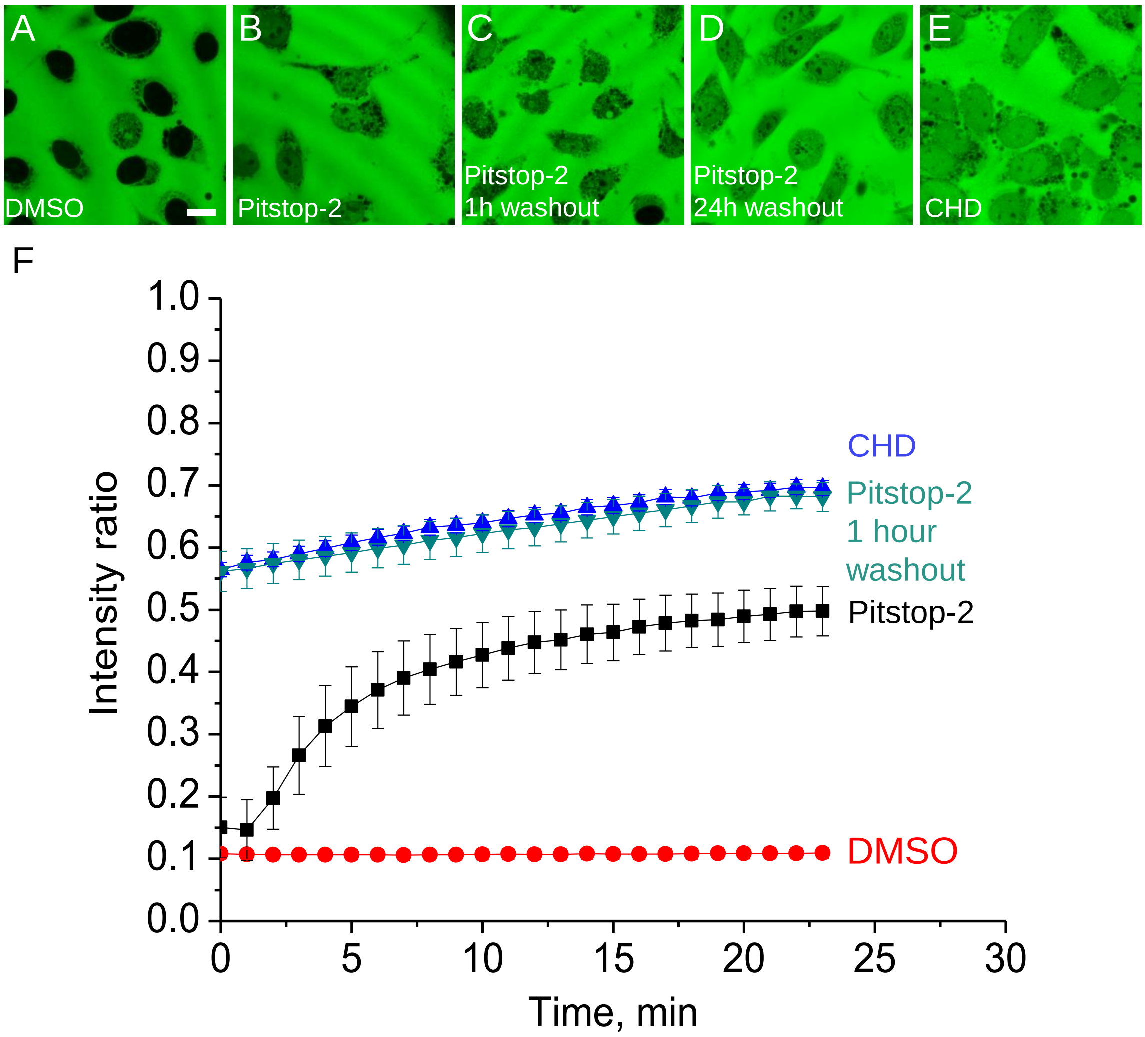

Figure S4|Pitstop-2 induces collapse of the NPC permeability barrier. Representative confocal images of digitonin-permeabilized Ea.hy 926 cells at 30 minutes after treatment with DMSO **(A)**, Pitstop-2 **(B)** and CHD **(E)**. Washout of Pitstop-2 one hour **(C)** or 24 hours **(D)** prior to permeabilization does not lead to restoration of permeability barrier in this particular cell line. Kinetics of 70kDa dextran influx into the nuclei of digitonin-permeabilized cells upon treatment with respective compounds **(F)**. Scale bar = 10µm. 20 cells from three separate experiments were analyzed for each condition. Error bars represent standard error of the mean.

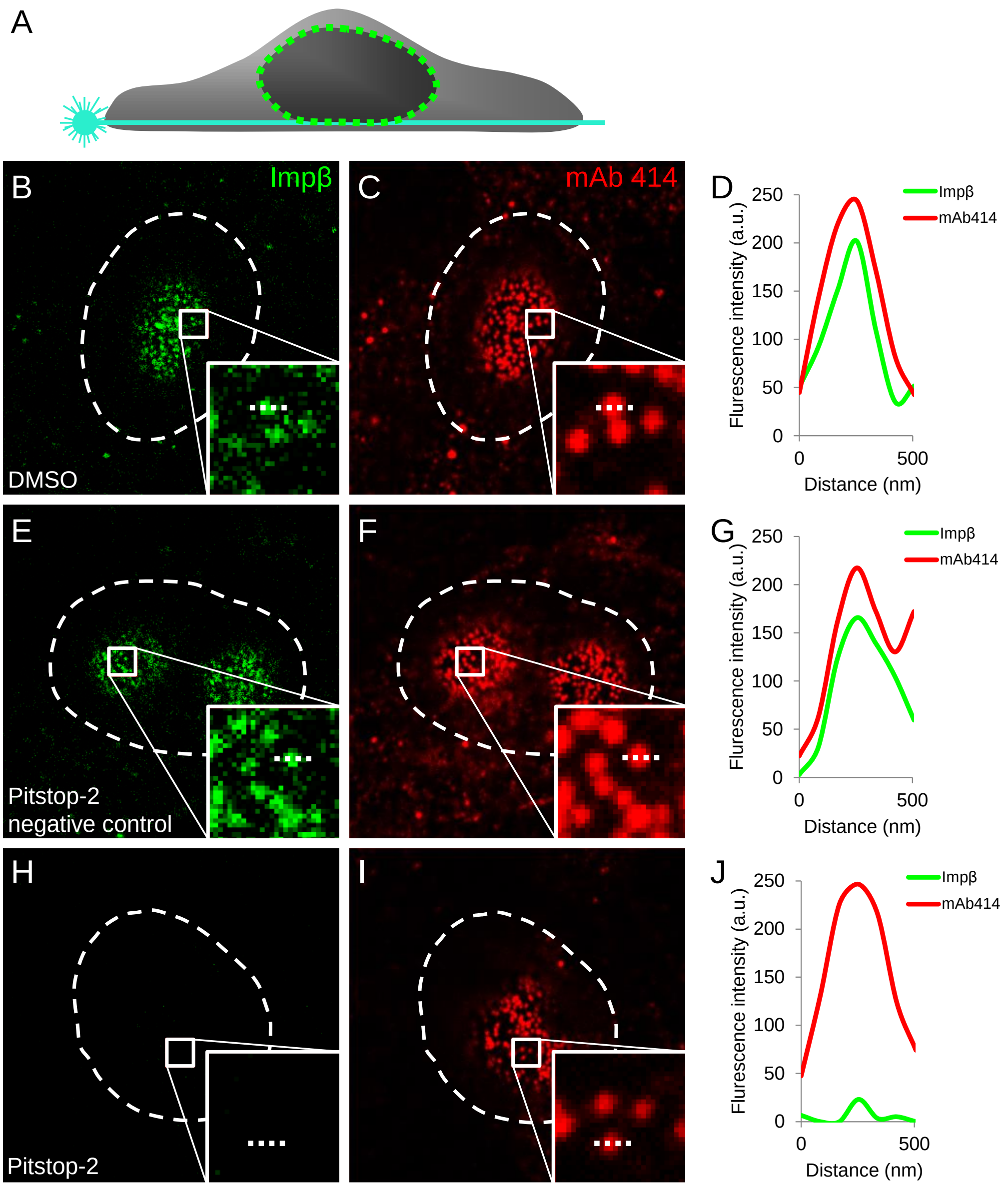

Figure S5|Quantification of Impβ-mAb414 colocalization. Schematic representation of the confocal image acquisition used to acquire images for ImpB-mAb414 colocalization analysis. The lower surface of the nucleus closest to the glass was used for the analysis **(A)**. Representative images of cells stained with ImpB and mAb414 and treated with DMSO **(B, C)**, Pitstop-2 negative control **(E, F)** and Pitstop-2 **(H, I)**. Dashed line in large image panels represents the outline of the nucleus. Dashed lines in the magnified insets are the intensity sections plotted in panels **(D, G, J)**. Each large image panel is 25x25µm, insets are 2x2µm.

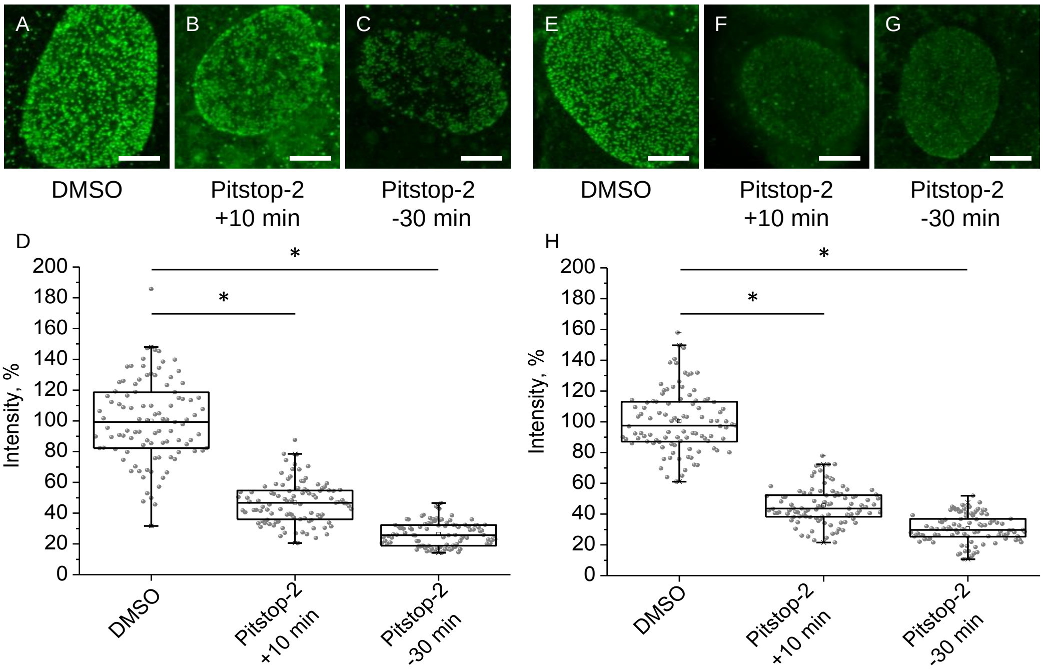

Figure S6|Pitstop-2 impairs binding of Impβ to the NPCs. Impβ binding to NPCs of GM 7373 cells **(A-C)** and Ea.Hy926 cells **(E-G)** at indicated time-points of Pitstop-2 addition. Permeabilization and Impβ addition is set as timepoint = 0 min. Scale bar = 5µm. Statistical analysis of the Impβ binding to NPCs of GM7373 cells **(D)** and Ea.Hy926 cells **(H)** treated with Pitstop-2. 100 individual NPCs from 20 different cells were analyzed for each condition. Statistical significance of the differences was assessed by Mann-Whitney test at $p \leq 0.05$.

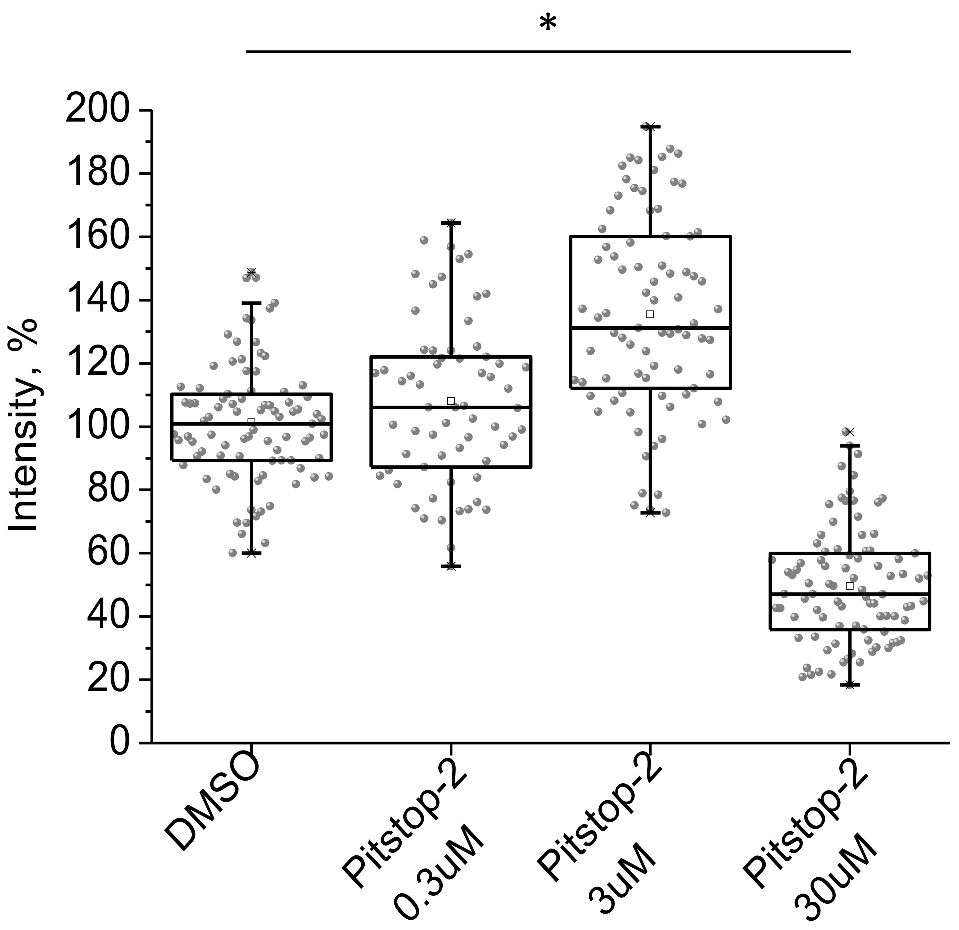

Figure S7| Pitstop-2 inhibits Impβ binding to the NPCs in a concentration dependent manner. Effect of three 10-fold dilutions of Pitstop-2 on Impβ binding to the NPCs of Ea.Hy926 human endothelial cells was tested. 30μM Pitstop-2 demonstrated the most robust inhibition of the Impβ binding to the NPCs.

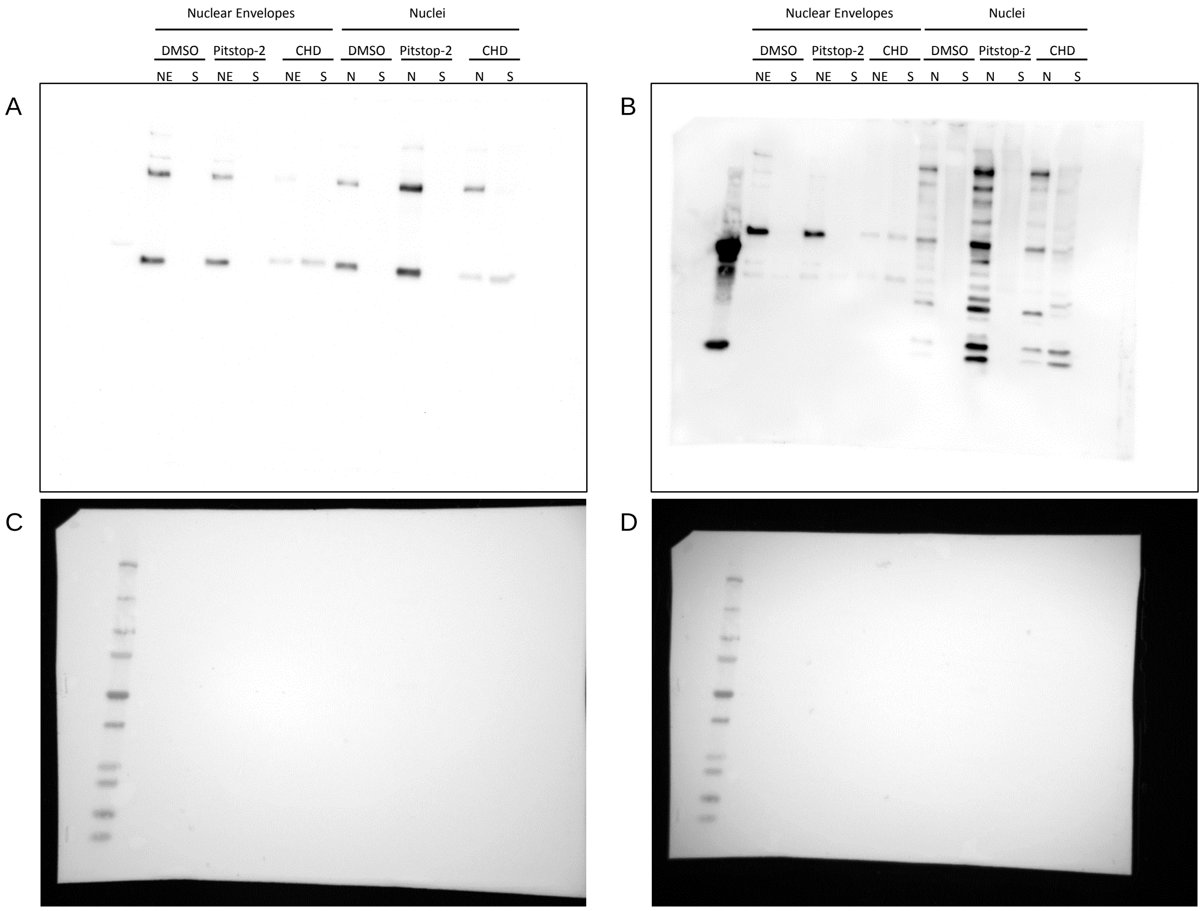

Figure S8|Pitstop-2 does not dissociate candidate permeability barrier-forming FG-Nups from the NPCs. Nuclear envelopes and nuclei were extracted with DMSO, Pitstop-2 and CHD and corresponding extracts were probed with mAB414 (full size blot scan) **(A)**. The same blot stripped and reprobed with anti-Nup98 antibody **(B)**. Corresponding images of blots imaged by epi-illumination with white light for molecular weight reference **(C, D)**.

A

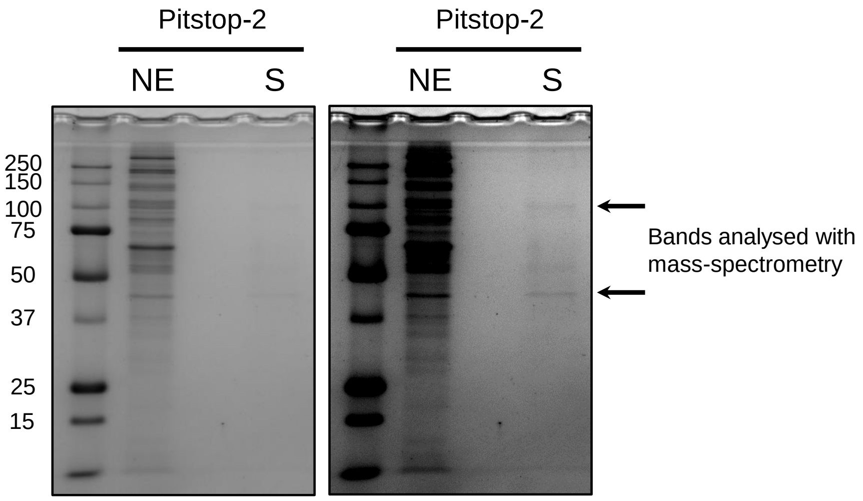

B

Entry	Description
TERA_XENLA	Transitional endoplasmic reticulum ATPase OS Xenopus laevis GN vcp PE 1 SV 3
HIBN_XENLA	Histone binding protein N1 N2 OS Xenopus laevis PE 1 SV 3
K2C8_XENLA	Keratin type II cytoskeletal 8 OS Xenopus laevis PE 2 SV 1
MCM5A_XENLA	DNA replication licensing factor mcm5 A OS Xenopus laevis GN mcm5 a PE 1 SV 2
IBTK_XENLA	Inhibitor of Bruton tyrosine kinase OS Xenopus laevis GN ibtk PE 2 SV 1
MCM3M_XENLA	Maternal DNA replication licensing factor mcm3 OS Xenopus laevis GN mmcm3 PE 1 SV 2
R7BP_XENLA	Regulator of G protein signaling 7 binding protein OS Xenopus laevis GN rgs7bp PE 2 SV 1
KC1G1_XENLA	Casein kinase I isoform gamma 1 OS Xenopus laevis GN csnk1g1 PE 2 SV 1
MCM6M_XENLA	Maternal DNA replication licensing factor mcm6 OS Xenopus laevis GN mmcm6 PE 1 SV 1
HNDLB_XENLA	Heterogeneous nuclear ribonucleoprotein D like B OS Xenopus laevis GN hnrpdl b PE 2 SV 1
CUL3A_XENLA	Cullin 3 A OS Xenopus laevis GN cul3a PE 1 SV 1
NT5D3_XENLA	NT5D3_XENLA
Q2VPH0_XENLA	MGC132155 protein OS Xenopus laevis GN MGC132155 PE 2 SV 1
Q6DDS8_XENLA	N1 N2 protein OS Xenopus laevis GN N1 N2 PE 2 SV 1
A2BDA9_XENLA	LOC100037087 protein OS Xenopus laevis GN LOC100037087 PE 2 SV 1
B1WB22_XENTR	Krt8 2 protein OS Xenopus tropicalis GN krt8 2 PE 2 SV 1
Q5XG33_XENLA	LOC495224 protein OS Xenopus laevis GN efs PE 2 SV 1

Figure S9|Pitstop-2 does not dissociate Nups from the NPCs. SDS-PAGE of the nuclear envelope (NE) and the supernatant (S) fraction of 100 *X. laevis* nuclear envelopes extracted with 30µM Pitstop-2 does not reveal any prominent bands in the supernatant fraction even after overexposure (right). Bands marked with the arrows were analyzed by mass spectrometry **(A)**. A list of proteins detected in the analyzed bands. No nuclear pore complex proteins were detected **(B)**.
